# A meat- or dairy-based complementary diet leads to distinct growth patterns in formula-fed infants: a randomized controlled trial

**DOI:** 10.1093/ajcn/nqy038

**Published:** 2018-04-20

**Authors:** Minghua Tang, Audrey E Hendricks, Nancy F Krebs

**Affiliations:** 1 Section of Nutrition, Department of Pediatrics, University of Colorado School of Medicine, Aurora, CO; 2 Department of Mathematical and Statistical Science, University of Colorado Denver, Denver, CO; 3 Department of Biostatistics and Bioinformatics, Colorado School of Public Health, University of Colorado Anschutz Medical Campus, Aurora, CO

**Keywords:** growth, infant, protein, complementary feeding, length for age, animal source foods

## Abstract

**Background:**

Protein intake from cow milk–based infant formula has been associated with rapid weight gain and increased adiposity, but the effect of protein from complementary foods has not been prospectively evaluated, and the effect of protein from sources other than formula during complementary feeding is not clear.

**Objective:**

The aim of this study was to directly compare the effect of protein from 2 common complementary food sources, meat and dairy, on infant growth and weight trajectory.

**Design:**

Healthy term, formula-fed infants were recruited from the metro Denver area, matched by sex and race/ethnicity and randomly assigned to a meat or a dairy complementary food group from 5 to 12 mo of age. Total protein intake during this 7-mo intervention was ∼3 g ⋅ kg^−1^ ⋅ d^−1^ for both groups. Intakes of infant formula, cereal, fruit, and vegetables were ad libitum. Caregivers also completed 3-d diet records at 5, 10, and 12 mo of age. Anthropometric measures were obtained during monthly home visits, and blood samples were collected at 5 and 12 mo of age.

**Results:**

Sixty-four infants completed the intervention (meat: *n* = 32; dairy: *n* = 32). The average total protein intake (mean ± SD) increased from 2.01 ± 0.06 g ⋅ kg^−1^ ⋅ d^−1^ at 5 mo to 3.35 ±0.12 g ⋅ kg^−1^ ⋅ d^−1^ at 12 mo and did not differ between groups. Over time, weight and weight-for-age *z* score increased by 0.48 ± 0.07. However, there was a significant group-by-time interaction for both length-for-age *z* score (LAZ) and weight-for-length *z* score (WLZ). Post hoc analysis showed that LAZ increased in the meat group (+0.33 ± 0.09; *P* = 0.001 over time) and decreased in the dairy group (−0.30 ± 0.10; *P* = 0.0002 over time); WLZ significantly increased in the dairy group (0.76 ± 0.21; *P* = 0.000002 over time) compared with the meat group (0.30 ± 0.17; *P* = 0.55 over time). Insulin-like growth factor I and insulin-like growth factor-binding protein 3 both increased over time without group differences.

**Conclusions:**

Protein source may have an important role in regulating growth. In these formula-fed older infants, meat- and dairy-based complementary foods led to distinct growth patterns, especially for length. This trial was registered at www.clinicaltrials.gov as NCT02142647.

## INTRODUCTION

Evidence-based feeding guidance could yield long-term benefits for optimal growth and obesity prevention, especially early in life, when the rate of growth is high and may hold greater plasticity. Observational studies (1–3) have reported greater weight gain in formula-fed infants than in breastfed infants. Because standard cow milk–based formula has a higher protein content (∼2.2 g protein/100 kcal) than breast milk (∼1.5 g protein/100 kcal), the higher protein intake by formula-fed infants has been considered a key contributor to the greater weight gain observed in formula-fed infants (4). Furthermore, a large-scale, multi-country intervention (5) compared isocaloric infant formula with high-protein (2.9 g/100 kcal) and low-protein (1.7 g/100 kcal) contents from birth to 12 mo. Results showed that weight gain was more rapid in the high-protein formula group, leading to a 0.20-SD higher weight-for-length *z* score (WLZ), a crude parameter of overweight status, but linear growth or length-for-age *z* score (LAZ) did not differ between groups. Another smaller study (6) also found that infants fed a high-protein formula (2.7 g/100 kcal) gained more weight than did infants fed a low-protein formula (1.65 g/kcal) from 3 to 6 mo of age, with no impact on linear growth. These findings have been the basis of recent recommendations to limit protein intake to ≤15% of total energy intake from 0 to 2 y of age to mitigate the risk of overweight and adiposity (7). By the nature of the studies, the focus was primarily on protein from a dairy source (i.e., infant formula). However, the effects of protein from other sources on infant weight gain and linear growth are not well studied.

Complementary feeding, when solid or semi-solid foods are added to infants’ diets in addition to human milk or formula, usually starts at 5–6 mo of age. During this time, other sources of protein become available to the infants. Effects of protein from complementary foods on infant growth have not been well studied and have primarily focused on breastfed infants. One study conducted by our research group examined the effect of meat as the main source of protein in complementary foods on growth in breastfed infants (8). Results showed that a high-protein, meat-based complementary diet (total protein: 2.7 g ⋅ kg^−1^ ⋅ d^−1^) increased both LAZ and weight-for-age *z* score (WAZ), compared with the low-protein, cereal-based diet (total protein: 1 g ⋅ kg^−1^ ⋅ d^−1^), without significantly changing WLZ. These findings suggest that a meat-based complementary diet may promote linear growth (LAZ) in breastfed infants without increasing overweight risks (WLZ). However, these findings cannot be directly applied to formula-fed infants who consume a different, higher-protein liquid diet (i.e., formula) and may respond differently to the same complementary foods. Moreover, the effects of dairy proteins for complementary foods on infants’ growth, especially formula-fed infants who are already at high risk of excessive weight gain, warrant further investigation. The current limited complementary feeding recommendations generally do not separate breastfed from formula-fed infants (7). Research on complementary feeding and optimal growth is urgently needed to make evidence-based recommendations. Thus, the purpose of the present study is to directly compare meat with dairy as the main sources of protein during complementary feeding on infant growth and weight trajectory in formula-fed infants. We hypothesized that, compared with dairy protein, meat would promote linear growth without increasing overweight risks in infants.

## METHODS

### Study design

This study was a stratified randomized controlled trial utilizing semi-controlled feeding. Infant formula and meat- or dairy-based complementary foods were provided for 7 mo (5–12 mo of age). The primary outcome of this study was growth, including longitudinal changes in weight (kilograms), length (centimeters), and their respective age- and sex-specific *z* scores. Secondary outcomes were blood biomarkers, including insulin-like growth factor I (IGF-I), insulin-like growth factor-binding protein 3 (IGFBP3), and blood urea nitrogen (BUN). Infant growth was measured at baseline and at 7, 8, 9, 10, 11, and 12 mo of age. Blood biomarkers were measured at baseline and at 12 mo of age. Upon recruitment to the study, participants were matched to another participant with the use of 10 race/ethnicity categories. The treatment assignment for the first participant in each matched pair was randomly assigned in Microsoft Excel. The matched participant for each pair was automatically assigned to the opposite treatment group. After stratifying by sex and ethnicity, 5-mo-old, exclusively formula-fed infants (≤1 mo of cumulative breastfeeding) were randomly assigned to receive either puréed meats or dairy foods, such as infant yogurt, cheese, or whey protein powder. Participants visited the Pediatric Clinical and Translational Research Center (CTRC) at Children's Hospital Colorado at baseline (5 mo) and at the end of the intervention (12 mo). Both visits included blood, 3-d diet record, and anthropometric measures. Anthropometric measurements (length, weight, head circumference) were also measured every month during the intervention at home visits. Compliance and health history were also obtained at the monthly home visits. This intervention was not blinded to the participants or the research team, except for the CTRC nurses who assessed anthropometric measurements and drew blood at baseline and at the end of the intervention. This study was approved by the Colorado Multiple Institutional Review Board and was registered at clinicaltrials.gov (NCT02142647).

### Participants

Term, formula-fed infants were recruited from the metro Denver area with the use of mail-in flyers by the Colorado Department of Public Health, which had access to birth records and could target mailings to households with a 3- to 5-mo-old infant. Parents who were interested in participating called the number on the flyer and completed a phone screening with the study coordinator. If eligible, a baseline visit at Children's Hospital Colorado was scheduled. After consenting, participants were randomly assigned to meat or dairy as the main complementary food protein, while matching for sex and ethnicity. Only exclusively formula-fed infants were chosen *1*) to increase internal validity because breast- and formula-fed infants pose different risks to rapid weight gain and may respond differently to complementary feeding, *2*) because formula-fed infants are at higher risk of excessive weight gain, and *3*) because the majority of infants in the United States are formula-fed, especially after 3 mo of age (9). Exclusion criteria included low birth weight, cumulative breastfeeding >1 mo, and significant congenital anomalies or known chronic diseases. Infant demographic data included sex, gestational age, birth weight, and brief medical history. Maternal and family medical history, parental weight status, and mode of delivery were also obtained.

### Dietary intervention and monitoring

During the 5- to 12-mo dietary intervention, total protein intake, including formula, was targeted at ∼3 g ⋅ kg^−1^ ⋅ d^−1^ (8), a quantity used in a previous feeding trial in older breastfed infants by our research team. In addition, information available at the time indicated that median and mean protein intakes for 6- to 11-mo-old infants were 19 and 22 g/d (10). Infants consumed a standard, intact milk protein–based formula ad libitum. Infant formula (Similac Optigro; Abbott Nutrition) was provided during the intervention to standardize this exposure. The meat-based diet consisted of commercially available puréed meats, and the dairy-based diet consisted of infant yogurt, cheese, and a powdered concentrate of 80% whey protein (specially packaged for this study by Leprino Foods). Whey protein was provided to augment total protein intake and to balance the casein-to-whey ratio. It has been used to treat malnutrition and stunting in low-resource settings due to its putative growth-promoting features. Fruit and vegetable intakes were not restricted. Parents were provided with tailored feeding guidelines and were encouraged to let the infant's appetite dictate his or her total intake, as done with previous complementary feeding interventions by our group (11–13).

At baseline and each home visit, parents were asked to estimate how much formula the infant was consuming daily, on average, over the past 2 wk. This number was used to calculate grams of protein from formula. On the basis of the body weight recorded at the visit, a recommendation with regard to an appropriate amount of meat- or dairy-based food was given to the parents in order to approximate a total protein intake of ∼ 3 g ⋅ kg^−1^ ⋅ d^−1^ (**Table 1**). Parents were also given a monthly calendar to record the daily consumption of formula and protein-based foods. At the end of each month, a 3-d diet record was completed and picked up at the monthly home visit, together with the calendar. The research coordinator reviewed the diet record and the food calendar with the parents and answered any questions or concerns. Parents also returned the leftover unused foods and formula, if any, to the study coordinator at the home visits as a crude validation that they were using the products provided. Three 3-d diet records (de-identified) at 5, 10, and 12 mo were analyzed by the CTRC Nutrition Core (NDSR software).

**TABLE 1:** Example of dietary intakes of a reference 9-mo-old female with a body weight of 8.5 kg (∼60th percentile weight-for-age) for the 2 feeding groups^1^

	Protein,	Energy,
Food item^2^	g/d	kcal/d
Liquid diet		
Formula: 20 ounces^3^ (not restricted)	10	480
Dairy-based complementary foods		
One infant yogurt	5	80
One cheese stick	8	90
Whey protein, 2.5 g	2	10
Meat-based complementary foods		
One jar of commercially puréed ham and gravy	8	70
One jar of commercially puréed beef and gravy	8	70

^1^Formula, fruit, and vegetable intakes were not restricted or controlled.

^2^Based on an estimated total calorie intake of 700 kcal/d (14) and a total protein intake of 3 g ⋅ kg^−1^ ⋅ d^−1^ (25.5 g/d, 102 kcal/d)

^3^1 ounce = 30 mL.

### Anthropometric measurements

Length, weight, and head circumference were measured at 5 and 12 mo by the research nurses at the CTRC who were blinded to the infants’ feeding group. Measurements were also obtained at each interim monthly home visit (6, 7, 8, 9, 10, and 11 mo). All measurements were performed in triplicate by trained research personnel. Length was measured in the recumbent position by using an infant stadiometer accurate to 0.1 cm (Holtain Ltd.). An electronic digital balance (Sartorious Corp.) was used to obtain naked infant weight. *z* Scores were calculated on the basis of WHO/CDC growth standards (15).

### Sample collection and analyses

Blood samples were collected at baseline and at the end of the intervention. Samples sit at room temperature for 30 min and were centrifuged at 1500 × *g* for 10 min and serum was stored at −80°C until analysis. The following markers were analyzed by the Colorado Clinical and Translational Science Institute's Core Lab: IGF-I (chemiluminescence; DiaSorin Liaison), IGFBP3 (chemiluminescence; Siemen), and BUN. The between-assay precisions were <2.7% for IGF-I, <4.0% for IGFBP3, and <4.5% for BUN.

### Statistical approach

On the basis of previous research by our group (8), we expected a minimum difference of ΔLAZ from 5 to 12 mo between the meat and dairy groups of 0.4, considering an SD of the difference between the 2 sample means of 0.4 and an α = 0.05. We planned to have ≥30 infants in each group to complete the intervention (power >90%). The recruitment goal was *n* = 75 total to account for a 20% drop-out rate.

Statistical analyses were performed with the use of SAS (version 9.3; SAS Institute). All model assumptions were checked before conducting the analysis. Group data are presented as means ± SDs. Baseline variables were compared with the use of independent Student's *t* test between the meat and dairy groups. For categorical variables, chi-square tests were used. Repeated-measures ANOVA (PROC GLM) was used to evaluate the main effects of time, group, and their interactions on the dependent variables. Maternal height and BMI were included in the model as covariates. Student's *t* test was used as a post hoc analysis to compare values between groups at each time point (paired), changes over time between groups (independent), and changes over time within each group (paired). One-sample *t* test was used to compare the weight and length velocities with the WHO standard. All model assumptions were checked, and *P* < 0.05 was considered significant.

## RESULTS

### Participant characteristics

**FIGURE 1:**
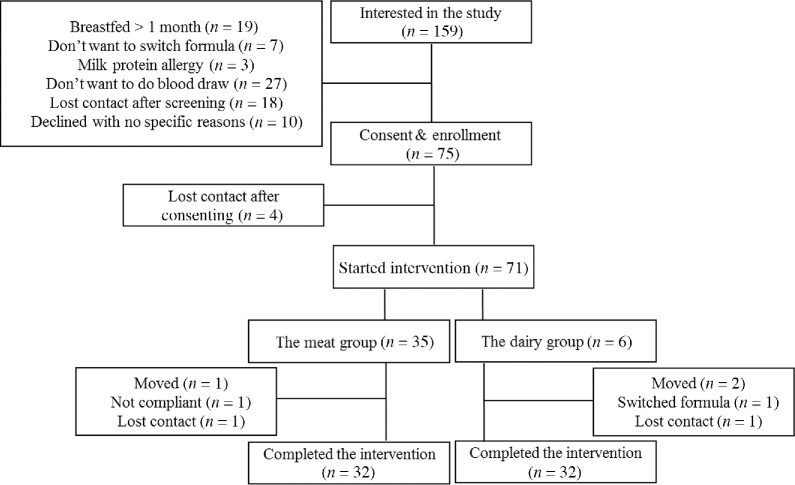
CONSORT diagram. CONSORT, CONsolidated Standards Of Reporting Trials.

Recruitment and screening started in September 2013, and the trial was completed in August 2016. A total of 159 infants were screened and 75 were enrolled in the study (**Figure 1**). Primary reasons for ineligibility included cumulative breastfeeding >1 mo and unwillingness to undergo blood draw. Overall, 64 infants (meat group: *n* = 32; dairy group: *n* = 32) completed the intervention between October 2014 and August 2016. Of the 11 infants who did not complete the study, 4 did not start the intervention after signing the consent form, 3 moved out of state during the intervention, 2 lost contact, 1 was not compliant, and 1 wanted to switch formula. Baseline characteristics are summarized in **Table 2**. Because participants were matched for race and sex, there was no difference in these variables between groups. On average, mothers of the participants were considered overweight [BMI (kg/m^2^) > 25] without differences between groups. Participants’ morbidity, including antibiotics intake, was also monitored every month during the intervention, and there was no difference between groups in terms of morbidity.

**TABLE 2:** Participant baseline characteristics^1^

	Meat (*n* = 32)	Dairy (*n* = 32)	*P*
Male sex, %	45	48	0.55^2^
Race and ethnicity, %	75, white; 19, Hispanic; 3, black; 3, Asian	75, white; 16, Hispanic; 6, black; 3 Asian	0.88^2^
Birth weight, kg	3.31 ± 0.37	3.33 ± 0.48	0.85^3^
Gestational age, wk	39 ± 1	39 ± 1	0.30^3^
Maternal BMI, kg/m2	28 ± 7	27 ± 6	0.45^3^
Maternal height, cm	167 ± 7	165 ± 8	0.32^3^
Maternal age, y	30 ± 6	29 ± 7	0.56^3^

^1^Values are means ± SDs unless otherwise indicated. Dairy, dairy-based complementary protein group; Meat, meat-based complementary protein group.

^2^Determined by chi-square test.

^3^Determined by independent Student's *t* test.

### Dietary intake

**FIGURE 2:**
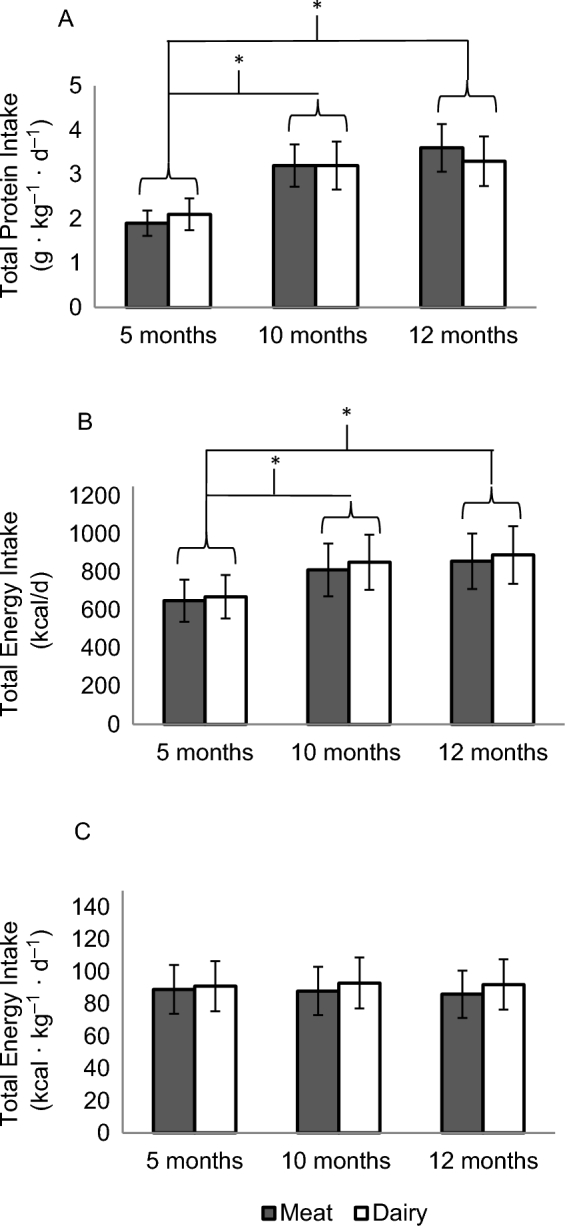
Total protein (A) and energy (B, C) intakes (means ± SDs) at 5, 10, and 12 mo of age. Repeated-measures ANOVA of time and group (meat compared with dairy: *n* = 32 compared with *n* = 32). Total protein (A) and total energy (B) intakes increased from baseline to 10 and 12 mo. (A) *Group-by-time interaction, *P* = 0.66; main effect of time, *P* < 0.001. (B) *Group-by-time interaction, *P* = 0.53; main effect of time, *P* < 0.005. There was no difference in protein intake between 10 and 12 mo or between groups at any time points. (C) Total energy intake did not change over time or differ between groups. Dairy, dairy-based complementary protein group; Meat, meat-based complementary protein group.

During screening at 5 mo of age, parents were asked whether the infant had started complementary foods. The majority of the participants were either still exclusively formula fed (52%) or only consumed cereal and fruit or vegetable purées (43%). Only 4% of the participants had tasted yogurt before the intervention, but none consumed yogurt on a regular basis, and none consumed meat. Total protein or energy intakes did not differ significantly between groups at baseline (5 mo) or at 10 or 12 mo of age. At baseline, protein intake was 2.01 ± 0.45 and 2.02 ± 0.58 g ⋅ kg^−1^ ⋅ d^−1^ for the meat and dairy groups, respectively. As expected, protein intake increased to a little over 3 g ⋅ kg^−1^ ⋅ d^−1^ at 10 and 12 mo (**Figure 2**). Total energy intake (kilocalories per day) also increased over time without significant differences between groups; energy intake per kilogram of body weight did not change (Figure 2). Complementary food intakes gradually increased over time, as expected. For example, protein intake from formula accounted for an average of 78% of total protein at baseline. This number decreased to 33% and 20% at 10 and 12 mo, respectively, without differences between groups at any time point of measure. Energy from protein (percentage of total energy) was 9% ± 1%, 15% ± 3%, and 15% ± 4% at baseline and at 10 and 12 mo, respectively. There were no significant differences in total fat, saturated fat, or total carbohydrate intakes between groups at any time point (data not shown).

Total protein intake was further broken down to sources including formula, meat, dairy, and vegetable proteins. Overall, consumption of formula decreased and solid foods increased over time. During the intervention, the majority of protein from solid foods came from the provided meat- or dairy-based foods, and the meat and dairy groups consumed primarily meat- and dairy-based solid foods, respectively. For example, at 10 mo of age, participants from the meat group consumed, on average, 2.1 g protein ⋅ kg^−1^ ⋅ d^−1^ from meat-based solid foods and only 0.03 g ⋅ kg^−1^ ⋅ d^−1^ from dairy-based solid foods, whereas participants from the dairy group consumed 2.0 g protein ⋅ kg^−1^ ⋅ d^−1^ from dairy-based solid foods and 0.01 g protein ⋅ kg^−1^ ⋅ d^−1^ from meat-based solid foods. Similar results were found at 12 mo of age (data not shown). In terms of essential amino acids (grams per day), there was no difference between groups at baseline. At 12 mo, the meat group consumed a significantly higher total amount of isoleucine (meat compared with dairy: 1.86 ± 0.31 compared with 1.55 ± 0.35 g/d; *P* = 0.03), lysine (meat compared with dairy: 2.92 ± 0.61 compared with 2.14 ± 0.58 g/d; *P* = 0.001), methionine (meat compared with dairy: 0.93 ± 0.33 compared with 0.68 ± 0.16 g/d; *P* = 0.001), and histidine (meat compared with dairy: 1.12 ± 0.24 compared with 0.78 ± 0.16 g/d; *P* = 0.0002); intakes of other amino acids were not significantly different.

### Growth

**FIGURE 3:**
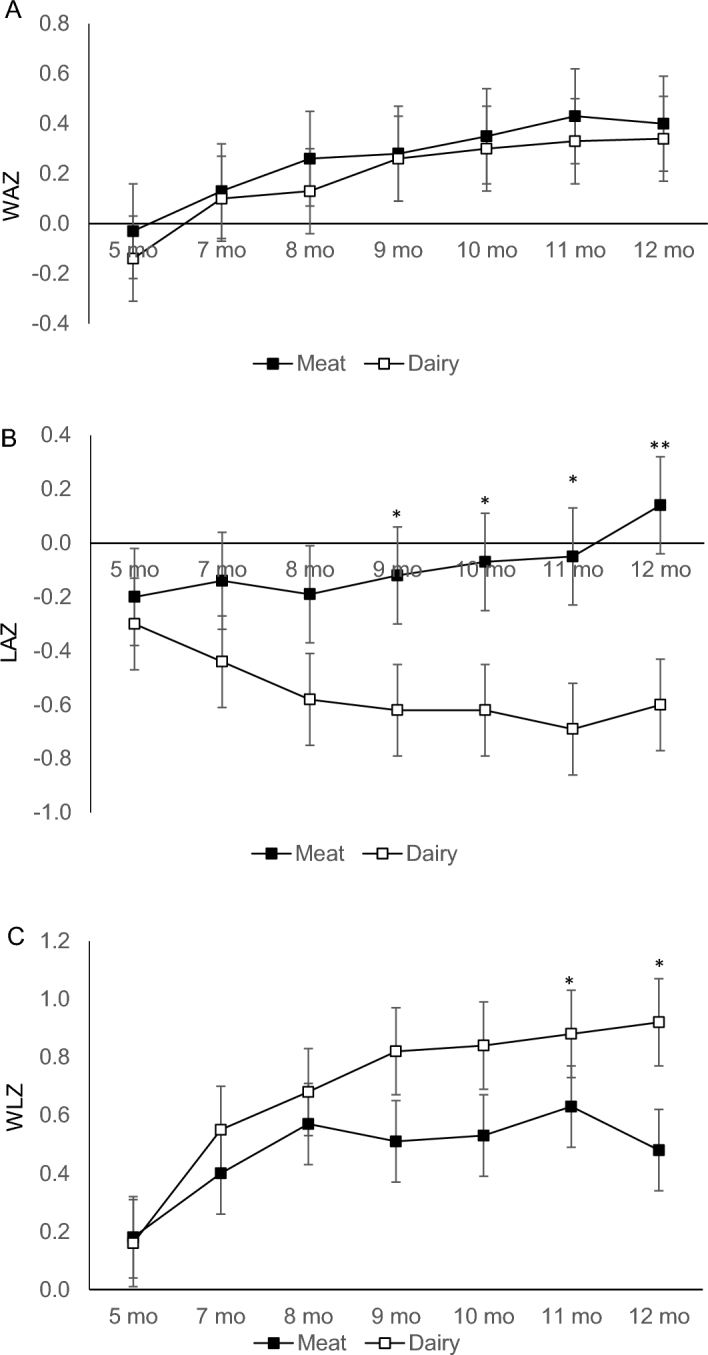
WAZ (A), LAZ (B), and WLZ (C) at baseline and at 7, 8, 9, 10, 11, and 12 mo of age (mean ± SD). Repeated-measures ANOVA of time and group (Meat compared with Dairy: *n* = 32 compared with *n* = 32) with maternal BMI and height as covariates. (A) Group-by-time interaction, *P* = 0.49. There was a significant main effect of time only (*P* = 0.0006). (B) Significant group-by-time interaction, *P* = 0.00001. LAZ differed between groups at 9, 10, 11, and 12 mo of age: *P < 0.05, **P = 0.001. (C) Significant group-by-time interaction, *P* = 0.015. WLZ differed between groups at 11 and 12 mo of age, *P = 0.03. Dairy, dairy-based complementary protein group; LAZ, length-for-age *z* score; Meat, meat-based complementary protein group; WAZ, weight-for-age *z* score; WLZ, weight-for-length *z* score.

Weight and length velocities, expressed as 2-mo gram and centimeter increments by sex, were compared with the WHO standards (16) and are summarized in **Tables 3** and **4**. There was no difference by sex or group in terms of weight or length velocity. Most weight increments were higher than the WHO median. *z* Scores (WAZ, LAZ, and WLZ) are summarized in **Table 5** and **Figure 3**. There was no difference in weight, length, head circumference, or *z* scores between groups at 5 mo. The average WAZ and LAZ were below the WHO median at baseline for both groups, and WLZs were above the WHO median. WAZ increased over time without a significant difference between groups at any time point (effect of time: *P* = 0.0006; group-by-time interaction: *P* = 0.49). A significant group-by-time interaction (*P* = 0.00001) was found for LAZ: LAZ increased (0.33 ± 0.09) in the meat group and decreased (−0.30 ± 0.10) in the dairy group. Significant differences in LAZ between groups emerged at 9 mo and continued at 10, 11, and 12 mo (Figure 3). At 12 mo, the average difference of length between groups was 0.74 SDs. The changes in WAZ and LAZ led to a significant group-by-time interaction (*P* = 0.015) for WLZ. Post hoc analysis of this significant interaction used paired *t* test to compare changes over time within each group. Results showed that WLZ significantly increased in the dairy group only (0.76 ± 0.21) and not the meat group (0.30 ± 0.17). WLZ was significantly higher in the dairy group compared with the meat group at 12 mo, and the average difference between groups at 12 mo was 0.44 (*P* = 0.03). There was no significant change in head circumference *z* scores during the intervention (Table 5). When including maternal height and BMI as covariates in the models, the significance of findings was not affected, except for a slight change in *P* values: WAZ effect of time, *P* = 0.001; LAZ group-by-time interaction, *P* = 0.0003; and WLZ group-by-time interaction, *P* = 0.019. The inclusion of partial data from participants who did not complete the study also did not affect the primary findings.

**TABLE 3:** Weight velocity (grams) at 2-mo increments between groups and the WHO median.^1^

	Male infants, g/2 mo			Female infants, g/2 mo		
Age	Meat	Dairy	WHO	Meat	Dairy	WHO
5–7 mo	730 ± 367	740 ± 324	778	798 ± 367	741 ± 327	742
7–9 mo	817 ± 190*	750 ± 192*	601	786 ± 197*	898 ± 245*	581
8–10 mo	636 ± 189*	693 ± 205*	544	670 ± 239	708 ± 228*	517
9–11 mo	635 ± 283*	650 ± 218*	502	666 ± 248*	583 ± 245	478
10–12 mo	626 ± 358	753 ± 412*	478	629 ± 269*	621 ± 336*	458

^1^Values are means ± SDs unless otherwise indicated. There was no difference between male and female infants or the meat and dairy groups at any time points. *Different from the WHO length velocity, *P* < 0.05. Dairy, dairy-based complementary protein group; Meat, meat-based complementary protein group.

**TABLE 4:** Length velocity (centimeters) at 2-mo increments between groups and the WHO median^1^

	Male infants, cm/2 mo			Female infants, cm/2 mo		
Age	Meat	Dairy	WHO	Meat	Dairy	WHO
5–7 mo	2.6 ± 1.1*	1.6 ± 1.3*	3.2	2.8 ± 1.5	2.4 ± 1.5*	3.2
7–9 mo	2.9 ± 0.9	2.8 ± 1.4	2.8	3.1 ± 0.9	2.6 ± 1.4	2.9
8–10 mo	2.8 ± 1.1	2.7 ± 1.1	2.7	3.0 ± 1.2	2.8 ± 1.3	2.7
9–11 mo	2.7 ± 0.8	2.4 ± 1.0	2.6	2.8 ± 0.9	2.6 ± 1.1	2.6
10–12 mo	2.9 ± 1.1	2.8 ± 1.5	2.5	3.5 ± 1.3*	2.7 ± 1.5	2.5

^1^Values are means ± SDs unless otherwise indicated. There was no difference between male and female infants or the meat and dairy groups at any time points. *Different from the WHO weight velocity, *P* < 0.05. Dairy, dairy-based complementary protein group; Meat, meat-based complementary protein group.

**TABLE 5:** Anthropometric measurements at 5 and 12 mo of age^1^

	5 mo		12 mo		
	Meat	Dairy	Meat	Dairy	Group-by-time interaction^2^
Weight, kg	7.37 ± 0.67	7.35 ± 0.74	9.92 ± 0.91	9.92 ± 0.97	0.81
Length, cm	65.3 ± 2.2	65.3 ± 2.5	75.7 ± 2.6	73.9 ± 2.2	0.00002
Head circumference, cm	43.0 ± 1.2	43.1 ± 1.1	46.4 ± 1.3	46.3 ± 1.1	0.55
Head circumference, *z* score	0.51 ± 0.82	0.54 ± 0.77	0.55 ± 0.81	0.49 ± 0.79	0.41

^1^Values are means ± SDs; *n* = 32 for the meat-based protein group and *n* = 32 for the dairy-based protein group. Dairy, dairy-based complementary protein group; Meat, meat-based complementary protein group.

^2^Repeated-measures ANOVA for group (meat compared with dairy) and time.

### Serum biomarkers

**Table 6** shows IGF-I, IGFBP3, and BUN concentrations between groups at 5 and 12 mo. Both IGF-I and IGFBP3 increased over time but remained within the normal range, without significant differences between groups. BUN increased over time (*P* = 0.001) without significant group differences and still within the normal range for this age (7–26 mg/dL). BUN was used to crudely evaluate protein intake over time for compliance. The increase in BUN over time is consistent with the increase in protein intake (Figure 2).

**TABLE 6:** IGF-I, IGFBP3, and BUN concentrations at 5 and 12 mo of age^1^

	5 mo		12 mo		*P*	
	Meat	Dairy	Meat	Dairy	Group-by-time interaction	Main effect of time
IGF-I, ng/mL	66 ± 20	61 ± 17	77 ± 27	70 ± 25	0.62	0.007
IGFBP3, ng/mL	2261 ± 420	2165 ± 421	2468 ± 545	2532 ± 532	0.89	0.00003
BUN, mg/dL	9 ± 2	8 ± 2	14 ± 5	15 ± 5	0.32	<0.00001

^1^Values are means ± SDs; *n* = 30 for the meat-based protein group and *n* = 31 for the dairy-based protein group. BUN, blood urea nitrogen; Dairy, dairy-based complementary protein group; IGF-I, insulin-like growth factor I; IGFBP3, insulin-like growth factor-binding protein 3; Meat, meat-based complementary protein group.

## DISCUSSION

To our knowledge, this is the first randomized controlled trial in formula-fed infants that directly compared the effects of dietary protein from 2 common complementary foods on infant growth trajectory and relevant serum biomarkers. The intervention did not seem to affect intakes, because both the meat and dairy groups reported similar amount of total calories, protein, and fat; only the main sources of protein were different between groups. The main finding of this study is that, in addition to the protein quantity, as previous research showed, protein source also had a significant impact on infant growth during the first year of life. Compared with dairy-based complementary foods of approximately the same quantity, meat-based complementary foods promoted linear growth (i.e., increased LAZ from below to above the WHO median). Compared with the WHO median, which is based on breastfed infants, weight velocity was significantly higher in both meat and dairy groups. This pattern of weight gain was expected for formula-fed infants, because previous research has shown that formula-fed infants gain at a faster rate than breastfed infants (1, 17).

Previous research also showed mixed results of length trajectory in relation to breast- compared with formula feeding: some studies found greater length and LAZ in formula-fed infants (2, 3), whereas others did not (18). Compared with the WHO median, length velocity in our study was significantly higher in the meat group from 10 to 12 mo and lower in the dairy group from 5 to 7 mo. As Figure 3 shows, LAZ in the dairy group tended to progressively deviate downward from the WHO median over the 7-mo intervention, whereas LAZ in the meat group slightly increased from below to above the median. The reference breastfeeding cohort in the study by Koletzko et al. (5) found that LAZ was relatively stable in breastfed infants from 6 to 12 mo of age, although total protein intake was not reported. A previous cohort of breastfed infants from studies in Denver by our research team showed that consuming a meat-based complementary diet (2.7 g total protein ⋅ kg^−1^ ⋅ d^−1^) increased LAZ by an average of 0.27, whereas a low-protein, cereal-based diet (1 g protein ⋅ kg^−1^ ⋅ d^−1^) decreased LAZ by an average of 0.33 over 4 mo (5–9 mo of age) (9). In the present study, the dairy group showed a similar decline in LAZ (−0.30) over 7 mo (**Supplemental Figure 1**). Consensus holds that the growth pattern of breastfed infants is the gold standard. Thus, it is unclear whether this LAZ decline in the dairy group, similar to that in our previous breastfed cohort (19), represents a harmful effect or not. The decline of LAZ in the dairy group was unexpected in a cohort who consumes infant formula and relatively high quantities of calories and protein; rather, LAZ was expected to remain the same or increase, as seen in the meat group. Although the protein intake of our previous Denver breastfed infant cohort (19) had a slightly higher percentage of energy from protein (17%), the energy intake for both groups in the current study was >30% higher than that of the breastfed cohort.

In terms of overweight risk, WLZ at 12 mo was 0.44 SDs higher for the dairy group compared with that of the meat group. This significant difference between groups was primarily driven by the difference in length or LAZ. Increasing WLZ across centiles on the growth chart is usually considered a warning for increased risk of becoming overweight (20). A “normal” growth pattern for infants and toddlers would be that WLZ remains relatively stable over time. In our study, the WLZ increase in the dairy group was 0.76 ± 0.39 SDs over 7 mo (**Supplemental Table 1**), suggesting excessive weight gain relative to length gain and a possible increased risk of becoming overweight over a short period of time. Koletzko et al. (5) showed an average WLZ increase of 0.89 from birth to 24 mo in the high-protein formula group and a WLZ difference of 0.20 between the high- and low-protein formula groups at 24 mo, controlling only the protein quantity in infant formula. In our study, the protein content in infant formula and total protein intake were essentially the same between groups, but the main source of protein from complementary foods differed. Similar findings of WLZ changes between the present study and the study by Koletzko et al. (5), within a much shorter intervention period, strongly highlight the importance of complementary food protein quality for infant growth.

Recent feeding guidelines from Europe recommend limiting protein intake early in life to a maximum of 15% of total energy (7). In our study, total protein intake increased from ∼2 g ⋅ kg^−1^ ⋅ d^−1^ at baseline to a little over 3 g ⋅ kg^−1^ ⋅ d^−1^, which was equivalent to 15% of energy from protein. This quantity of protein may also have contributed to the linear growth promotion observed in the meat group. The Feeding Infants and Toddlers Study (FITS) (10) showed that the median protein intake in 6- to 11-mo-old US infants was 9% and the 90th percentile was 13%. In addition, the 2009–2012 NHANES data showed that 6- to 11-mo-old US infants consume a mean protein intake of 2.4 g ⋅ kg^−1^ ⋅ d^−1^, with the 75th and 90th percentiles at 3 and 4 g ⋅ kg^−1^ ⋅ d^−1^ (21). However, the consumption of baby food meats declined from 2002 to 2008 by 80% in older infants, without compensating for increases in other protein sources, whereas yogurt consumption increased in younger toddlers (22). These observations emphasize the need for evidence-based recommendations to guide complementary feeding practice in order to foster optimal growth.

Although IGF-I was considered the key mediator of WLZ increase in the study by Koletzko et al. (5), it is also the most copious growth factor in human bone (23) and greatly influences linear growth (e.g., LAZ). Both animal (24) and human (25) studies have shown that IGF-I stimulates bone growth. A longitudinal observational trial found that formula-fed infants had higher IGF-I concentrations than did breastfed infants, which was positively associated with both weight gain and length gain from birth to 3 mo of age (26). In our study, IGF-I and IGFBP3 increased over time in both groups and was positively associated with the increases in WAZ and LAZ in the meat group. This was expected because IGF-I secretion is responsive to protein intake and the secretions of IGF-I and IGFBP3 usually mimic each other (27). However, why the dairy group had relatively decelerated linear growth is not clear and cannot be explained by the change in IGF-I and IGFBP3 per se. Branched-chain amino acids may also be associated with the promotion of linear growth and weight gain in both animal (28) and human (29) models. Thus, it is possible that the higher intake of isoleucine in the meat group may have contributed to the observed greater linear growth. Further mechanistic investigations are warranted, such as analyzing blood amino acid profiles and metabolomics analysis.

One of the strengths of the study was that the reported protein intakes during the intervention accurately reflected the randomization, with no difference in total energy or other macronutrient consumption. In addition, before randomization, participants were matched by sex and race to reduce potential confounding factors. There were also a few limitations of the study. First, we were not able to blind the study coordinator or the parents because the complementary foods provided could be easily identified as meat- or dairy-based. This could potentially bias the anthropometric outcomes. However, the CTRC research nurses who measured baseline and end-of-study anthropometric variables were blinded to group assignment. Another potential bias was that no formal standardization sessions were conducted for this study to document inter- and intra-anthropometrist precision and reliability. Second, WLZ was used to assess overweight risks and adiposity, but it is not a direct measure of body composition. Third, due to funding constraints, we did not include a reference breastfeeding group to directly compare the 2 intervention groups of formula-fed infants. However, the 2 intervention groups were compared with the WHO standards, which are based on breastfed infants and with previously studied local breastfed cohorts (Supplemental Figure 1). However, we do not believe this limitation influences the strength of the findings for formula-fed infants. Fourth, although distinct growth patterns were observed based on different protein sources, whether this pattern would persist in the long term still needs to be investigated. Fifth, the 3-d diet records completed by parents may not accurately reflect the actual intakes of the participants, although we have no basis to conclude that the inaccuracies of diet records would differ between groups. Finally, we did not follow the 2 participants who did not complete the intervention due to not being compliant and switching formula. This was a violation of the intent-to-treat principle.

In summary, our findings suggest that protein intakes from complementary foods can significantly affect infant growth and possibly overweight risks. Furthermore, protein sources may be as important as protein quantity in terms of growth regulation. Meat and dairy as high-quality proteins are commonly consumed in infants’ diets, and yet we found that they led to distinct growth patterns in these formula-fed infants. However, it is unclear whether the increase in LAZ in the meat group would have any health benefits in well-nourished infants. The current Dietary Guidelines for Americans has very limited guidance for infants and children from birth to 24 mo. Findings from our study reinforce the need for more high-quality research on dietary patterns and nutrient intakes in infants in relation to the quality of growth.

## Supplementary Material

Supplemental data

## Notes

Supported by NIH (National Institute of Diabetes and Digestive and Kidney Diseases) 1K01DK111665, NIH/National Center for Advancing Translational Sciences (NCATS) Colorado Clinical and Translational Science Awards (CTSA) grant UL1 TR001082, and (alphabetically) Abbott Nutrition, the American Heart Association, the Beef Checkoff through the National Cattlemen's Beef Association, Leprino Foods, and the National Pork Board.

Supplemental Figure 1 and Supplemental Table 1 are available from the “Supplementary data” link in the online posting of the article and from the same link in the online table of contents at https://academic.oup.com/ajcn/.

Abbreviations used: BUN, blood urea nitrogen; CTRC, Pediatric Clinical and Translational Research Center; IGF-I, insulin-like growth factor I; IGFBP3, insulin-like growth factor-binding protein 3; LAZ, length-for-age *z* score; WAZ, weight-for-age *z* score; WLZ: weight-for-length *z* score.
