# Supplementary material for: A meat- or dairy-based complementary diet leads to distinct growth patterns in formula-fed infants: a randomized controlled trial
Source: Am J Clin Nutr. 2018 Apr 20;107(5):734–42. doi: 10.1093/ajcn/nqy038 (PMC6128676; doi:10.1093/ajcn/nqy038)
Supplement: Supplemental data [file nqy038_supp.zip › ajcn163360-file001.docx]

**Supplementary figure. Z scores (WAZ, LAZ, WLZ) of the current study and the previous Denver cohort of breastfed infants^1^**

^1^Tang M, Krebs NF. High protein intake from meat as complementary food increases growth but not adiposity in breastfed infants: a randomized trial. Am J Clin Nutr. 2014;100(5):1322-8. Epub 2014/10/22. doi: 10.3945/ajcn.114.088807. PubMed PMID: 25332329; PMCID: 4196483.
