# Supplementary material for: A meat- or dairy-based complementary diet leads to distinct growth patterns in formula-fed infants: a randomized controlled trial
Source: Am J Clin Nutr. 2018 Apr 20;107(5):734–42. doi: 10.1093/ajcn/nqy038 (PMC6128676; doi:10.1093/ajcn/nqy038)
Supplement: Supplemental data [file nqy038_supp.zip › ajcn163360-file002.docx]

**Supplementary Table 1**. **Anthropometric measurements during the intervention^1^**

|  | 5 months | | 7 months | | 8 months | | 9 months | | 10 months | | 11 months | | 12 months | |
| --- | --- | --- | --- | --- | --- | --- | --- | --- | --- | --- | --- | --- | --- | --- |
|  | Meat^2^ | Dairy^2^ | Meat^2^ | Dairy^2^ | Meat^2^ | Dairy^2^ | Meat^2^ | Dairy^2^ | Meat^2^ | Dairy^2^ | Meat^2^ | Dairy^2^ | Meat^2^ | Dairy^2^ |
| Weight (kg) | 7.37 ± 0.67 | 7.35 ± 0.74 | 8.17 ± 0.70 | 8.10 ± 0.77 | 8.63 ± 0.78 | 8.49 ± 0.74 | 8.90 ± 0.75 | 8.91 ± 0.75 | 9.28 ± 0.78 | 9.23 ± 0.78 | 9.61 ± 0.81 | 9.51 ± 0.79 | 9.92 ±0.91 | 9.92 ± 0.97 |
| Length (cm) | 65.3 ± 2.2 | 65.3 ± 2.5 | 68.1 ± 2.7 | 67.3± 2.2 | 69.5 ± 2.4 | 68.5 ± 2.3 | 71.0 ± 2.3 | 69.9 ± 2.5 | 72.5 ± 2.6 | 71.2 ± 2.4 | 73.8 ± 2.6 | 72.3 ± 2.3 | 75.7 ± 2.6 | 73.9 ± 2.2 |
| WAZ | -0.03 ± 0.69 | -0.14 ± 0.82 | 0.13 ± 0.64 | 0.10 ± 0.72 | 0.26 ± 0.71 | 0.13 ± 0.72 | 0.28 ± 0.69 | 0.26 ± 0.70 | 0.35 ± 0.66 | 0.30 ± 0.71 | 0.43 ± 0.66 | 0.33 ± 0.69 | 0.40 ± 0.74 | 0.39 ± 0.78 |
| LAZ | -0.19 ± 0.86 | -0.30 ± 1.02 | -0.14 ± 1.02 | -0.44 ± 0.98 | -0.19 ± 0.88 | -0.58 ± 1.00 | -0.12 ± 0.86 | -0.62 ± 1.10 | -0.07 ± 0.91 | -0.62 ± 1.02 | -0.05 ± 0.91 | -0.69 ± 1.01 | 0.14 ± 0.90 | -0.60 ± 0.91 |
| WLZ | 0.18 ± 0.73 | 0.16 ± 0.88 | 0.40 ± 0.79 | 0.55 ± 0.95 | 0.57 ± 0.84 | 0.68 ± 0.94 | 0.51± 0.78 | 0.82 ± 0.96 | 0.53 ± 0.80 | 0.84 ± 0.98 | 0.63 ± 0.68 | 0.88 ± 0.88 | 0.48 ± 0.75 | 0.92 ± 0.88 |
| Head circumference Z | 0.51 ± 0.82 | 0.54 ± 0.77 | 0.36 ± 1.02 | 0.43 ± 0.80 | 0.45 ± 0.76 | 0.47± 0.95 | 0.36 ± 0.93 | 0.40 ± 0.90 | 0.48 ± 0.86 | 0.50 ± 0.90 | 0.38 ± 0.91 | 0.34 ± 0.76 | 0.55 ± 0.81 | 0.49 ± 0.79 |

^1^Mean ± SD

^2^Meat: the meat-based complementary diet group (n=32); Dairy: the dairy-based complementary diet group (n=32)
